# About *Chlamydia trachomatis*. Reply to Garcia-Teillard et al. Trachoma and the Importance of Sexual Infective Route in Developed Countries. Comment on “Gallenga et al. Why the SAFE—*S* Strategy for Trachoma? Are *Musca sorbens* or *Scatophaga stercoraria* Really the Culprit?—A Brief Historical Review from an Italian Point of View. *Pathogens* 2023, *12*, 1419”

**DOI:** 10.3390/pathogens13050414

**Published:** 2024-05-16

**Authors:** Martina Maritati, Carlo Contini, Marco Del Boccio, Rossella D’Aloisio, Pio Conti, Marco Mura, Pier Enrico Gallenga, Carla Enrica Gallenga

**Affiliations:** 1 Department of Translational Medicine, University of Ferrara, 44124 Ferrara, Italy; marco.mura@unife.it; 2 Department of Medical Sciences, University of Ferrara, 44124 Ferrara, Italy; cnc@unife.it; 3 Centro Medico Santa Lucia, 66054 Vasto, Italy; gilbertodelboccio2019@gmail.com; 4 Department of Medical, Oral, and Biotechnological Sciences, G. d’Annunzio University Chieti-Pescara, 66100 Chieti, Italy; oftalmologia@unich.it; 5 Molecular Immunopharmacology and Drug Discovery Laboratory, Tufts University Medical School, Boston, MA 02111, USA; pioconti@yahoo.it; 6 Stenella cno Ophthalmology Laboratory, 65100 Pescara, Italy; studiogallenga@libero.it; 7 Bioethical Committee of San Marino Republic, 47893 Borgo Maggiore, San Marino; 8 Department of Medical Sciences, University of Ferrara, Eye Clinic University-Hospital, 44124 Ferrara, Italy; gllcln@unife.it

The confirmatory comment of Garcia-Teillard et al. [1] has led us to formulate an answer based on three points:

- The treatment of infection with targeted and prolonged antibiotic therapy that can interfere with the *Chlamydia trachomatis* life cycle, reducing antibiotic inappropriateness and, thus, efficacy;
- Reducing indiscriminate pesticide pollution that interferes with the food chain and reduces biodiversity;
- Evaluating the active role of dipterans in the transmission of infection in urban areas by raising awareness of sexually transmitted infection among Health Authorities through information and prevention, differentiating between protection and contraception, with a current increase in the spread of infection in the competent population.

The comment by Damian Garcia-Teillard et al. [1] regarding the suggestion about the need to increase awareness of *Chlamydia trachomatis* (*Ct*) infection due to the sexual infectious route [2] helps to pave the way for more articulated answers and considerations.

**First: On the subject of antibiotic treatment of Ct**

There are official papers edited by the Federation of European Ophthalmology-(FEOph) symposia aimed at standardizing the consensus for the administration of antibiotics for prophylaxis and treatment [3]. It is necessary to highlight that this is important especially in high-income countries, whereas in low-income countries, where the active trachoma is endemic, the mass drug administration of antibiotics is a key strategy in the eradication attempt [4].

It is well known that the conjunctival epithelial cells can become infected by the *Ct* serovars D through K, which cause genital diseases [5]. The pathogenesis of this process is determined by direct contact with infected genital secretions, resulting in sexually acquired chlamydial conjunctivitis. This conjunctivitis presents as a non-purulent erythematous injection of the epithelial surface with a pebbled look [6] that could mimic acute allergic follicular conjunctivitis, resulting in an underestimated diagnosis. Therefore, in non-endemic areas for trachoma, the differential diagnosis is an important step in the diagnostic process. In this regard, calling the attention of Health Authorities to control the spread of the disease by providing more information on prevention in high schools in high-income countries (market surveys document a post-COVID 19 reduction in condom consumption and persistent confusion between protection and oral contraceptives, especially in adolescents) is another priority for the WHO. 

*Chlamydia trachomatis* has also been detected in the human oral cavity [7]. Consequently, chlamydial infectious bodies from a patient’s pharynx might contribute to *Ct* transmission [8].

Other important questions that still need to be answered are related to the understanding of the mechanisms of *Ct* clinical persistence as *Ct* can trigger pelvic inflammation leading to chronic disease and infertility as well as cancer with or without the presence of co-pathogens [9], and reducing the resistance development and consumption effectiveness of new generations of antibiotics, but also of old and well-established antibiotics such as tetracycline, in different clinical studies [10].

**Second: Role of pesticide pollution**

It is mandatory to reduce pesticide pollution. Pesticides are widely used against insects in agriculture and against mosquitoes and flies in urban environments. 

Pesticide pollution can also affect rural areas; with rain, pesticides wash into rivers, reducing the survival of fish and causing the fertilization of algae at the marine mouths of rivers. To clarify the scenario, it is necessary to highlight that the so-called regulation on the sustainable use of plant protection products (Sur) was presented in June 2022 to the European Parliament, with the ambitious goal of halving pesticides by 2030 [11] to reduce the impacts of pesticides on human health and the environment. But at the moment, the opportunity has been missed.

Garcia-Teillard et al. rightly report the different habitats of insects in rural communities in comparison to the sanitation of urban areas (E stands for “Environment” in the acronym SAFE, the WHO strategy for the eradication of trachoma) [1].

It is also mandatory to evaluate the interference of the order Diptera with the rural anthropized environment, as in Figure 5 of the reference paper [2].

Thus, regarding the “dipteran identity problem”, [1] reports the article by Robinson et al. [12], which defines Musca sorbens as the main vector of specificity for human feces odor. Some light can be shone on the attitude of the species.

**Third: Role of dipterans in the transmission of infection in urban areas (*Musca sorbens*, *Scatophaga stercoraria* and related species)**

*Musca sorbens* (Species: M. sorbens, Order: Diptera, Class: Insecta), the bazaar fly or eye-seeking fly, is very similar in appearance to the housefly (*Musca domestica*), and it is the putative trachoma vector. *Musca sorbens* prefers human feces to reproduce, with the females being attracted by volatile semiochemicals and later transmitting the infection when feeding [13].

The yellow dung fly *Scathophaga stercoraria* (L.) (Diptera: Scathophagidae) is a widespread and locally abundant fly associated with the dung of large mammals. Although *S.stercoraria* is considered a cow dung specialist, it can successfully reproduce on the dung of other large mammals, such as sheep [14], horses, deer, or wild boar [15]. *Sarcophaga carnaria* (Diptera: Sarcophagidae) enters houses as it is attracted by the smells of the substrates on which the larvae feed.

Furthermore, it is essential to note that *Chlamydia* can remain viable in the intestine, paws, and proboscis for up to 6 hours and can contaminate different foods and objects with which people may come into contact [16].

In the adverse events/benefit ratio, the Authors’ suggestion [1] of an environmental control effort of Diptera using a “fly trap”, as suggested by Robinson et al. [12], appears to be very interesting and in line with the strategy for greater environmental safety.

Returning to *Ct*, Garcia-Teillard et al. correctly confirm how in some cities (except in rural areas with poor hygiene), the fly is an innocent vector; instead, transmission of the infection occurs through sexual intercourse or with hands soiled by contact with infected sexual areas, what the Authors call “the sexual infectious route” [1]. The Authors report a decline in sex education on sexually transmitted diseases and prevention methods, as mentioned earlier. Theres is an obvious confusion between “protection” (IULM University reports a decrease in condom sales [17]) and “contraceptives” (i.e., the “morning-after pill”: levonorgestrel) and a current increase in the spread of infections in the competent population. Therefore, improving intimate hygiene and sexual information will raise the level of awareness and prophylaxis.

We would like to thank the Authors for their attention and suggestions regarding our proposal to implement the WHO SAFE strategy with –S (SAFE—*S*) to draw attention to Sexual behavior.
